# Assessment of the implementation of the Italian National action plan to combact antimicrobial resistance (PNCAR) 2017–2021 through SPiNCAR-1: results for the Piedmont Region, 2022

**DOI:** 10.1186/s13756-025-01604-8

**Published:** 2025-07-09

**Authors:** Stefania Di Giacomo, Luca Bresciano, Lorenza Ferrara, Sabina Pederiva, Carla Maria Zotti, Fortunato “Paolo” D’Ancona, Costanza Vicentini, Collaborating Group, Roberto Alessi, Fabrizio Bert, Cesare Bolla, Francesco D’Aloia, Adriano De Simone, Gerolamo Farrauto, Scipione Gatti, Gianluigi Guano, Agostino Maiello, Barbara Mitola, Domenica Morabito, Federico Pagnoni, Luigi Raimondi, Maurizio Salvatico, Paola Silvaplana, Carlo Silvestre, Valentina Venturino, M. Rita Viroletti

**Affiliations:** 1 Department of Public Health and Pediatrics, University of Turin, Via Santena 5 Bis, 10126 Turin, Italy; 2 Epidemiology, Biostatistics and Mathematical Modeling Unit (EPI), Department of Infectious Diseases, Istituto Superiore Di Sanità (ISS), Viale Regina Elena, 299. 00161 Rome, Italy; 3 Settore “Prevenzione, Sanità Pubblica Veterinaria E Sicurezza Alimentare”, Regione Piemonte, Turin, Italy; 4 Servizio di Igiene e Sanità Pubblica, ASL AT, Asti, Italy

**Keywords:** Antimicrobial resistance, Self-assessment, Implementation, National plan to combat antimicrobial resistance, One health, Italy

## Abstract

**Background:**

The Italian National action plan to contrast antimicrobial resistance (PNCAR) was adopted in Italy with the aim of reducing antimicrobial resistance (AMR) rates through a “One Health” approach. The project “Support for the National Plan to Combat Antimicrobial Resistance (SPiNCAR)” was developed to evaluate the level of implementation of actions outlined by the PNCAR, through a self-assessment tool, addressed to regional and local health authorities. This study presents the findings of the first use of the SPiNCAR tool in the Region of Piedmont, detailing the level of implementation as of 31 December 2022.

**Methods:**

The self-assessment questionnaire is divided into 7 central "areas" representing the main actions against AMR: governance, surveillance and monitoring, appropriate use of antimicrobials, healthcare-associated infection (HAI) control and prevention, education and training, alliance among stakeholders, evaluation of the impact and implementation of the program. Areas are structured into two or more items called "standards", subdivided into "criteria”. Different questionnaires were developed for Regional and Local Authorities. Scores were calculated as the percentage of criteria met within each area, aggregated at both regional and local levels.

**Results:**

By analysing results at the regional level, it was possible to identify domains of strength in the areas of Governance (79%), Appropriate use of antimicrobials (70%), HAI Control and Prevention (68%) and areas for improvement in Alliance among Stakeholders (18%), Training (33%) and Surveillance (41%). Local authorities showed encouraging median results in Surveillance (37%), Appropriate Use of Antimicrobials (22,5%), and HAI Control and Prevention (21,5%), whereas Implementation (10,5%), Education and Training (8%) and Alliance among Stakeholders (2,5%) need to be improved.

**Conclusions:**

The first SPiNCAR assessment offered valuable insights to enhance healthcare quality; the evaluation identified stakeholder engagement and training as priority areas for improvement and targeted interventions for AMR containment.

**Supplementary Information:**

The online version contains supplementary material available at 10.1186/s13756-025-01604-8.

## Background

Antimicrobial resistance (AMR) is a major public health threat requiring intersectoral and multilevel actions.

Infections caused by AMR pathogens contribute significantly to the global disease burden: in 2019 roughly 1.27 million deaths were linked to AMR bacteria and this figure could increase to approximately 10 million deaths per year by 2050 if no effective actions are taken to address the issue [1] [2].

In Italy, the National Action Plan to Combat Antimicrobial Resistance (PNCAR), first introduced in 2017 and updated in 2022, sets forth a strategic, multidisciplinary framework based on a One Health approach. The plan emphasizes integrated governance and includes four key support areas: training; communication and transparency; research and bioethics; cooperation on national and international levels. It also addresses three primary intervention areas across human, animal, and environmental health: comprehensive monitoring of AMR, antibiotic use, and healthcare-associated infections (HAIs); HAI prevention in hospitals and communities; responsible antibiotic use and waste management. The PNCAR’s main goals are to improve HAI prevention and surveillance, promote appropriate antibiotic use in both human and veterinary contexts, advance research and innovation, raise public awareness, and strengthen cooperation domestically and internationally [3].

The "Support for the National Plan to Combat Antimicrobial Resistance (SPiNCAR)" project, carried out as part of the 2018 Centre for disease control and prevention (CCM) initiatives of the Italian Ministry of health, was created to implement the PNCAR 2017–2020 and its subsequent updates. The project led to the development of a self-assessment tool, SPiNCAR, which was settled with the collaboration of the Ministry of health, the National health institute (Istituto Superiore di Sanità, ISS), regional agencies, zooprophylactic institutes, local health authorities, and universities [4].

The SPiNCAR tool aims to provide Regions/Autonomous provinces (AP) and healthcare facilities with a common methodology to monitor the implementation status of the PNCAR. Through SPiNCAR, Regions/AP and healthcare facilities can continuously improve by evaluating their programs' strengths and weaknesses against uniform standards. Similarly, central institutions, particularly the Ministry of health and ISS, can access data showing the implementation status of actions outlined by the PNCAR [5].

SPiNCAR is a self-assessment system based on standardized indicators which allow the monitoring and evaluation of the actions against AMR implemented by Regions/AP and healthcare facilities, both public and private, affiliated, scientific institutes for research, hospitalization, and healthcare (“IRCSS”), both hospital and territorial, throughout the national territory, with a particular focus on public organizations. The monitoring focuses on actions taken to combat AMR in both human and veterinary fields, based on scientific evidence and the guidelines provided by the PNCAR[4].

By completing the framework, responders have the opportunity to assess how their actions align with a shared path of desirable and effective practices. This allows them to evaluate in detail the strengths and weaknesses of their implementation programs and to identify critical areas for improvement interventions.

The objective of this study is to present data from the Region of Piedmont analysing both regional and local results to identify disparities, areas of strengths and weaknesses, providing a possible model to other regions or nations seeking a tool to evaluate strategies against AMR.

## Methods

### SPiNCAR project – National level

The framework was established through a review of scientific literature and an analysis of available national and international guidelines and regulations on AMR and infection prevention and control (IPC), as previously described [6]. Initially, various intervention areas were identified, focusing on the most critical fields. For each area, a set of quality standards was defined, applicable to regional and local levels, highlighting the core elements needed to achieve the goals of the PNCAR. Each standard includes a set of essential and additional criteria, which correspond to minimum and supplementary performance levels, respectively. These criteria outline actions that need improvement, designed with increasing complexity step by focusing on basic issues before tackling more advanced challenges [6].

The development of the SPiNCAR self-assessment tool began with a comprehensive literature review to identify key domains and criteria relevant to AMR action plans. An initial draft of the questionnaire was then subjected to a Delphi consensus process. The Delphi method involved two rounds of online surveys with a panel of national experts in infectious diseases, public health, and epidemiology. Separate Delphi panels were convened for the regional and local versions of the questionnaire to ensure that each was tailored to the appropriate administrative level. After consensus was reached, pilot tests were conducted with regional and local health authorities. Participants completed the draft questionnaire and provided structured feedback on clarity, feasibility, and completion time. This feedback was used to make final revisions to the tool prior to its implementation [6].

The coordination of surveillance at national level is managed by ISS (Department of infectious diseases) in collaboration with the Directorate-General for health prevention of the Ministry of health.

For the purpose of the survey, the framework consists of a self-assessment questionnaire, which is available through a dedicated web platform (https://spincar.iss.it) [7]. The questionnaire covers seven thematic *areas*:

- *Governance*
- *Surveillance and monitoring*
- *Appropriate use of antimicrobials*
- *HAI control and prevention*
- *Education and Training*
- *Alliance among Stakeholders*
- *Evaluation of the impact and Implementation of the program*

Each area is set up of two or more *standards* corresponding to a set of action outlined in the PNCAR. Each action or requirement is called *criterion*. The criteria are divided into *minimum criteria* (mandatory for achieving the standard) and *additional criteria* (optional, not necessary for achieving the standard). The criteria are formulated as closed-ended questions (yes/no) and for each achieved criterion is given a unit score. The total score for a standard is made by summing all the scores of its minimum criteria and additional criteria separately. Additional criteria could be checked only if all the minimum criteria for that standard were met**.**

The areas incorporate criteria related to all involved health domains, following the One Health approach, not only limited to human health but also including animal health.

Italy has a universal public healthcare system known as *Servizio Sanitario Nazionale* (SSN). National health policies and priorities are under responsibility of the central government which determines annual SSN funding and controls the allocation of resources to each region. However the organization and delivery of health services is decentralized and entrusted to Regions, organized through Local health authorities (“Aziende Sanitarie Locali” or “ASL”) and Hospital trusts (“Aziende ospedaliere” or “AO”) [8]. Local health authorities are responsible for delivering comprehensive healthcare services within a specific geographic area [9]. Hospital trusts, on the other hand, focus mainly on secondary and tertiary care. Unlike Local health authorities, which manage a broader range of services, Hospital trusts concentrate specifically on hospital management and specialized care [8].

Different questionnaires were developed to evaluate both the regional level and the Local health authorities/Hospital trust level. The questionnaires for the regional level focus on the activities carried out by regional health authorities, which oversee the provision of healthcare within their respective territories. For the Local health authorities and Hospital trusts, separate questionnaires were designed to account for the differences in their organizational structures and roles within the healthcare system.

In May 2023, the Italian Ministry of health invited Regions/AP and healthcare facilities to carry out a first self-assessment through SPiNCAR concerning 2022 data; requested data had to refer to the situation as of 31 December 2022 and the compilation was carried out between June 15, 2023 and September 15, 2023. The Region of Piedmont, in Northern Italy, participated in the survey following ministerial indications.

### Study design, participation and analysis of results

This study reports results of the self-assessment performed in the Region of Piedmont in 2023, concerning the level of implementation of the PNCAR up to December 2022. As previously stated, the participation of regional health authorities of each Italian region was requested by the Ministry of health. Within Piedmont, all public trusts of the region were invited via official document to participate in SPiNCAR, as part of the regional indicator system for surveillance, prevention and control of HAIs and AMR. Participation was voluntary. The questionnaires were completed by a designated representative or infection control personnel within each medical direction. Completed questionnaires were submitted electronically through dedicated online platform [7]. Each authority was responsible for ensuring accuracy and completeness before submission. For the current study, we focused primarily on analysing the results of the framework's implementation at the regional level, with a smaller focus on the local level. First, we analysed data reported by the regional health authority of Piedmont. We calculated the percentage of minimum criteria observed out of the total available minimum criteria, and the percentage of additional criteria observed out of the total available additional criteria for each standard of each area. Subsequently, for each standard of each area, we calculated the percentage of both minimum and additional criteria met out of the total available minimum and additional criteria. Finally, for each standard in each area, we calculated the percentage of minimum criteria met out of the total available minimum and additional criteria. Scores of standards in the macro-areas were summarized using spider charts to visualize the performance of different indicators on a common scale. The total, or denominator, used to analyse the various "scores" of individual standards within each area is represented by the sum of all possible indicators present for that standard (including both minimum and additional). Furthermore, two overlapping charts were generated: orange spider chart illustrates the percentage scores representing the minimum criteria that should be met for each area. These are calculated as the ratio between the score achievable by meeting the minimum expected criteria and the total score available (from both minimum and additional criteria) for each standard. Each vertex of the polygonal area corresponds to a specific standard. The blue area, on the other hand, represents the percentage scores based on the actual performance of the region/trust. It is calculated as the ratio between the total score achieved (from both minimum and additional criteria) and the total possible score for each standard. Each vertex of the blue polygon corresponds to a standard.

This allows us to determine that if the blue area extends beyond the orange area, the minimum requirements for achieving the standard have been met, and that also additional (optional) criteria have been fulfilled. Conversely, when the blue area falls within the orange area, it means that the minimum criteria have not even been fully met. Second, results were assessed concerning the local level questionnaires. All public Local health authorities (n: 12), all Hospital trusts (n: 6) including teaching hospitals (n: 3) of the region completed the local-level SPiNCAR questionnaire. Spider charts were also used for the local level but using different parameters than the ones considered for regional level assessment: median scores were calculated for each standard, separately for Local health authorities and Hospital trusts; Therefore, the score of each facility was compared with the reference median (median of Local health authorities’ scores or median of Hospital trusts scores); results were summarized in a chart that was provided separately to each facility, offering personalized feedback on the results obtained.

## Results

### Regional level

#### Overall assessment

**Fig. 1:**
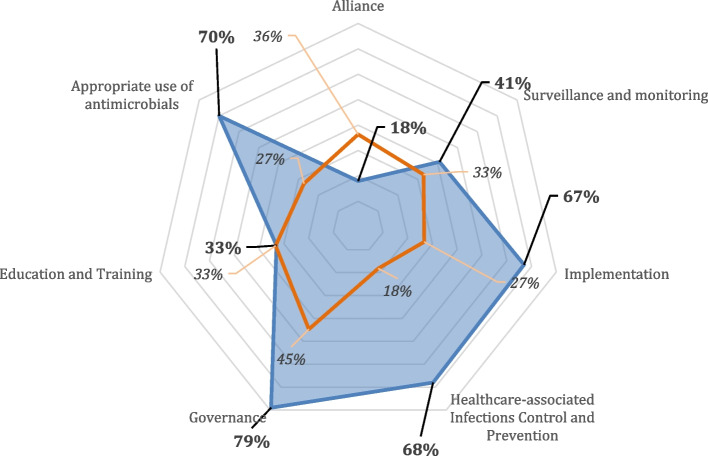
Graphical representation of regional level SPiNCAR scores achieved by the Region of Piedmont, Italy, 2022The radar charts represent the percentage score obtained considering the sum of standards for each area (in blue) compared to the minimum criteria that should be met for each area (in orange), expressed as a percentage

Table 1 and Fig. 1 summarize the overall results obtained by the region of Piedmont for each area. Results for standards within each area are available as Supplementary material.

**Table 1:** Regional level SPiNCAR scores achieved by the Region of Piedmont, Italy, 2022

Area	Score (%) **minimum criteria**	Score (%) **additional criteria**	Score (%) **total criteria**
*Governance*	94	67	79
*Surveillance and monitoring*	79	22	41
*Appropriate use of antimicrobials*	83	65	70
*Healthcare-associated Infections Control and Prevention*	100	61	68
*Education and Training*	60	20	33
*Alliance*	33	10	18
*Implementation*	100	55	67

### Governance area

The *Governance area* evaluates the capacity of the Piedmont Region to articulate and adopt a plan to contrast AMR based on a "One Health" approach, to identify targets and responsibilities, priorities and objectives. The minimum criteria amount to a total of 17, and all have been met except for one, namely: "The Region involves, in the Antimicrobial Resistance Control Plan, not only professionals operating in the healthcare sector but also those in the environmental and agricultural sectors, identifying their respective responsibilities". With regard to the additional criteria, 14 out of the 21 considered criteria have been met.

Consequently, a total score of 79% was achieved (30 minimum and additional criteria out of 38 total criteria).

### Surveillance and monitoring area

This area examines: the monitoring and surveillance measures adopted by the Region to report the spread of alert microorganisms; the presence of surveillance systems integrated with current national and international systems for monitoring antimicrobial resistance (through standardized reporting) and the consumption of antimicrobials in human, veterinary (livestock and companion animals), and agricultural settings; the monitoring of healthcare-associated infections (in regular wards and intensive care units).

The minimum criteria identified for this area are 29, of which 23 have been observed by the Piedmont Region. We observed the absence of a periodic analysis of data on the consumption of antimicrobials (at hospital and outpatient levels); monitoring the prevalence of HAIs in the context of outpatient residential facilities (e.g. LTCF) is implemented in less than 50% of the total facilities; the Region did not have a continuous monitoring system for resistance to antimicrobials in pets using the results of antimicrobial susceptibility testing (antibiograms) on clinical isolates produced by microbiological laboratories operating in the Region.

As regards the additional criteria, 13 of the 59 expected were observed. The overall score achieved (between minimum and additional criteria) for the surveillance area and monitoring is equal to 36 out of a total of 88 (41%).

### Appropriate use of antimicrobials area

In this context, it is evaluated the ability of Piedmont Region to adopt recommendations for managing infectious diseases and policies aimed at controlling antibiotic prescriptions and their appropriate use in both human and veterinary settings.

Over a total of 12 minimum criteria, 2 were not met: one refers to the requirement for public microbiology services to be open for at least 12 h a day and at least 5 days a week and the other about the adoption of a policy that ensures timely communication of positive culture results for multidrug resistance microorganisms by the microbiology service in all healthcare facilities. 21 out of the 32 additional expected criteria were met. Therefore, in the context of promoting a more appropriate use of antibiotics, a total score of 31 out of 44 criteria (70%) was achieved, considering both minimum and additional criteria.

### Healthcare-associated infections control and prevention area

In the context of controlling healthcare-associated infections (HAIs), the region's ability to implement a program for appropriate hand hygiene and a program for the implementation and monitoring of practices for prevention and control of HAI has been assessed. There are a total of 4 minimum criteria, all of which have been met. Regarding additional criteria, 11 out of the 18 specified criteria have been observed. Therefore, in the area of regional HAI control, a total score (including both minimum and additional criteria) of 15 out of 22 criteria (68%) has been achieved.

Some additional criteria were not met, specifically those concerning the involvement of private facilities in the program and the availability of regional guidelines for the adoption of related prevention and control measures.

### Education and training area

In this context, the region's competence to conduct training sessions is assessed, including specifying the necessary skill sets and providing courses for hospital staff, community workers, and veterinarians in both public and private sectors. These trainings cover topics such as antimicrobial resistance (AMR) and also surveillance, prevention, and control of healthcare-associated infections (HAIs).

There are a total of 10 minimum criteria, of which 6 have been met. The unmet criteria include promoting training on AMR and the appropriate use of antimicrobials for pharmacists and dentists working in public and private hospitals and outpatient settings through regional courses; promoting training for the surveillance, prevention, and control of HAIs for healthcare staffs and dentists working in public hospitals and outpatient settings, as well as in private facilities, through regional courses, offered both in-person and distance learning lessons.

Regarding additional criteria, 4 out of 20 have been observed. Consequently, in the area of training on the topics of the PNCAR, an overall score of 10 out of 32 criteria (33%) has been achieved.

### Alliance area

In this section, the adoption of information and training initiatives by the region is examined, focusing on AMR, the appropriate use of antimicrobials, and the prevention of HAIs. These initiatives are proposed to citizens and patients, farmers, and pet owners. Additionally, the active involvement of general practitioners, paediatricians, pharmacists, veterinarians, dentists, and the general population in combating AMR is also estimated.

Out of a total of 12 minimum criteria, only 4 have been met. The region has not provided informational materials to citizens/patients nor promoted activities in the last two years that involved active participation of these groups on the topics of AMR reduction and HAI prevention.

No information campaign about PNCAR matter targeting farmers and pet owners has been conducted. Additionally, in the last year, no initiatives have been undertaken to involve dentists or pharmacists working in the private sector in discussions about combating AMR, the appropriate use of antimicrobials, and the surveillance, prevention, and control of HAIs.

Furthermore, there is no indication of at least an annual meeting between the region and representatives of General Practitioners Associations/Orders and General Paediatricians to define and plan specific initiatives on the topics of AMR, the appropriate use of antimicrobials, and the surveillance, prevention, and control of HAIs.

The expected additional criteria were 21, only 2 of which were met. The overall score achieved (considering both minimum and additional criteria) for the area is 6 out of a total of 33 criteria (18%).

### Implementation area

In the context of assessing the impact and implementation of the program, the region's ability to annually evaluate the impact of the Regional Plan to combat antimicrobial resistance and disseminate the related data is analysed. There are a total of 4 minimum criteria, all of which were met. Regarding the additional criteria, 6 out of 11 were observed. Therefore, a total score (including both minimum and additional criteria) of 10 out of 15 actions implemented was achieved, reaching a percentage of 67%.

#### Local level

**Fig. 2:**
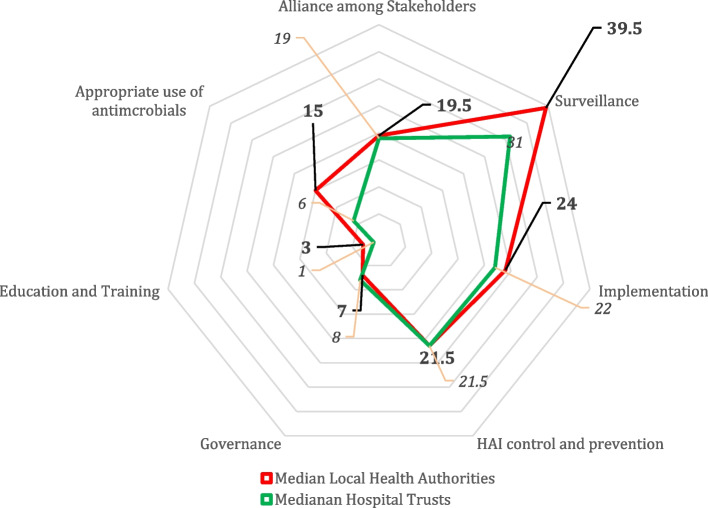
Graphical representation of local level SPiNCAR scores achieved in the Region of Piedmont, Italy, 2022. Median score of the Local health authorities (in red) and of Hospital trusts (in green) of the Piedmont Region obtained considering the sum of all standards for each area

All Local health authorities and all Hospital trusts of Piedmont completed the local-level questionnaire. The spider charts provided in Fig. 2 show the median value of the scores for each standard, calculated separately for all Local health authorities and for all Hospital trusts.

The Governance and Training areas result to be weak points both for Local health authorities and Hospital trusts, whereas the surveillance area represent a strong point.

## Discussion

This study reports the first experience of assessment through the SPiNCAR framework of the level of PNCAR implementation in the Piedmont Region, both at regional and local levels. The SPiNCAR tool provides a standardized methodology for self-assessment, allowing health organizations, at different levels, to compare their achievement with the national objectives outlined by the PNCAR.

According to results presented in this study, the governance area demonstrated strong capacity to adopt and articulate plans for fighting AMR using the “One Health” approach. With 94% of the minimum criteria achieved, the Piedmont Region showed a significant level of implementation.

This aligns with studies showing that effective governance is a cornerstone of successful AMR strategies. The global soft governance regime on AMR has led to widespread adoption of national action plans (NAPs) following the launch of the WHO's Global Action Plan in 2015. Most countries have begun implementing these plans, and implementation is associated with meaningful reductions in antibiotic consumption, especially in high-use countries. However, the effectiveness of implementation depends heavily on countries’ bureaucratic capacity. While a legally binding treaty is not deemed essential, it could help ensure sustained support and capacity-building for low-income countries [10]. In their study conducted between 2020–2021, Patel and colleagues developed and measured governance for 114 countries on the basis of an AMR governance framework. The global governance response to AMR remains modest and uneven, with an average score of 51/100 across 114 countries. While policy design and implementation tools show some progress, monitoring, evaluation, and accountability mechanisms are critically weak. Many countries lack clear responsibility assignments, public reporting, and stakeholder engagement. These governance gaps risk undermining the effectiveness of national AMR plans. Strengthening evaluation systems and global coordination is essential to align actions with the urgency of the AMR threat [11]*.*

Effective surveillance and monitoring systems are crucial for tracking AMR trends and advising policy choices. The performance of Piedmont in this area highlights strong data collection and reporting systems. This result could be explained by the fact that the Piedmont region introduced a performance indicator system in 2008 to allow an objective assessment of HAI surveillance, infection prevention and control activities, and structural and organizational resources.

The regional indicator system has promoted the participation in national surveillance systems such as the national surveillance for surgical site infection (Sistema Nazionale Sorveglianza Infezioni del Sito Chirurgico [SNICh]) [12] and the continuous surveillance in intensive care units through GiViTI (Gruppo Italiano per la Valutazione degli Interventi in Terapia Intensiva). Furthermore, Piedmont regularly takes part in point prevalence surveys of HAIs and antimicrobial use in acute-care hospitals and LTCFs [13] [14].

Furthermore, since 2014, in Piedmont hand hygiene practices are monitored as part of the regional indicator system [15] and from 2020 the region joined the national surveillance system for alcohol-based handrub consumption (Sorveglianza Nazionale Consumo Soluzione Idro-Alcolica [CSIA]), coordinated by ISS [16].

Stewardship programs are fundamental to guarantee the rational use of antimicrobials. In their systemic review and meta-analysis looking at how Antimicrobial Stewardship Programs (ASPs) are connected to antibiotic use, Ya et al. found that ASPs were linked to reduced antibiotic consumption in both hospitals and non-hospital settings and also significantly in paediatric patients [17]. Piedmont has implemented a number of initiatives to promote appropriate antibiotic use, however more could be done in such areas mainly as education and specific training for healthcare professionals [18].

The region has undertaken several IPC initiatives, such as developing locally adapted IPC protocols for both acute care and long-term care settings. Keeping high attention over hygiene practices and IPC measures will be necessary to maintain and improve what our region has achieved.

Education and Training healthcare professionals on AMR and IPC is essential. Postgraduate training, focused on continuous professional education, ensure that prescribers are aware of the latest international guidelines, understand and promote the importance of selecting the proper antibiotics molecules, dosing, and duration of treatment, and are capable to transfer stewardship principles in clinical practice. This approach has proved to be an effective and evidence-based method for reducing excessive inappropriate antibiotic use [19].

Piedmont's efforts in this area have been relevant, even if ongoing training and updates are needed to keep pace with evolving AMR challenges.

Building alliance among stakeholders, including healthcare providers, policymakers, and the public, is vital for a cohesive AMR strategy. Recent work underlines that growth and management of AMR should involve stakeholders from various sectors, including healthcare providers and veterinary medicine, environmental science, policymakers and more. The multidisciplinary nature of AMR needs to be handled bringing together different expertise and perspectives strategies, to minimize its impact as clinical burden through One Health approach [20].

Piedmont has fostered substantial collaborations, which should be strengthened and expanded.

The integration of professionals from environmental and agriculture sectors remains a critical area of improvement.

Regular assessment allows the identification of implementation gaps and to evaluate progress of both national and regional targets to reduce AMR prevalence. Evidence highlights the importance of monitoring antimicrobial use, resistance trends and the outcomes of ASPs to support best management practice. To achieve concrete improvement, it is recommended to use standardized metrics, establish surveillance systems, and uphold continued evaluation. These form the basis for evidence-based policymaking, efficient resource allocation, and for reaching AMR reduction [21].

This baseline assessment through SPiNCAR provides a valuable benchmark for ongoing assessments and strategic programming.

## Limitations

As highlighted by Bravo et al. the framework faces several limitations. Defining clear standards and criteria was not always possible, often due to objectives being too wide or with poor scientific evidence. The framework includes 264 and 279 criteria across seven areas, respectively for regional and local levels, making it time-consuming and requiring multiple stakeholders' involvement to accurately assess local and regional statuses [6]. Further, data at both regional and local levels were self-reported and a degree of understanding of the concepts and definitions underlying the SPiNCAR framework is required in order to accurately perform self-assessment. Therefore, we cannot exclude a degree of reporting bias or misunderstanding.

## Conclusions

The SPiNCAR framework has proven to be a useful tool for monitoring and evaluating the level of implementation of AMR control measures in the Piedmont Region. The comprehensive data collected through this self-assessment system highlights the region's strengths, such as high compliance with governance standards, effective surveillance systems, and different initiatives in antimicrobial stewardship and HAI control.

However, the analysis also reveals areas that require improvement, including the need for integration of environmental and agricultural sectors into AMR plans, better education and training programs for healthcare professionals, and stronger alliances among stakeholders to ensure a better approach to AMR. Looking forward, continuous improvement of AMR strategies based on SPiNCAR data is essential. Improving the framework to include more detailed criteria and comprehensive evaluations, along with increasing public awareness and education, will further support these initiatives.

## Supplementary Information

Supplementary Material 1.

## Data Availability

Datasets will be made available by the corresponding Author upon reasonable request.

## Abbreviations

AMR: Antimicrobial resistance
SPiNCAR: Supporto al Piano Nazionale di Contrasto all’Antimicrobico Resistenza
PNCAR: Piano Nazionale di Contrasto all’Antimicrobico Resistenza
ISS: Istituto Superiore di Sanità
HAI: Healthcare Associated Infection
CCM: Centro Nazionale per la Prevenzione ed il Controllo delle Malattie
AP: Autonomous Provinces
IRCSS: Istituti di Ricovero e Cura a Carattere Scientifico
SSN: Servizio Sanitario Nazionale
LTCF: Long-Term Care Facility
SNICh: Sorveglianza Infezioni del Sito Chirurgico
GiViTI: Gruppo Italiano per la Valutazione degli Interventi in Terapia Intensiva
NAP: National Action Plan
ASP: Antimicrobial Stewardship Program
CSIA: Consumo Soluzione Idro-Alcolica
