# Supplementary material for: Assessment of the implementation of the Italian National action plan to combact antimicrobial resistance (PNCAR) 2017–2021 through SPiNCAR-1: results for the Piedmont Region, 2022
Source: Antimicrob Resist Infect Control. 2025 Jul 9;14:86. doi: 10.1186/s13756-025-01604-8 (PMC12243208; doi:10.1186/s13756-025-01604-8)
Supplement: Supplementary file 1 — Supplementary Material 1. [file 13756_2025_1604_MOESM1_ESM.docx]

**Supplementary material**

***Figure 3. Governance area score, Piedmont Region 2022.***

*The spider chart shows the scores expressed as percentages (%) obtained for each individual evaluation standard* *within the Governance macro-area (corresponding to the vertices of the chart).*

*The blue area represents the scores (in bold) expressed as percentages (%) obtained from the sum of the criteria met by the Region (both minimum and additional) out of the total criteria (minimum and additional).
Example: Criterion 1.01: 84% = 16/19 x 100*

*The orange area represents the scores (in italics) expressed as percentages (%) that would have been achieved by meeting all the minimum criteria for each standard. This is calculated as the ratio of minimum criteria to the total number of criteria (minimum and additional). Example: Criterion 1.01: 37% = 7/19 x 100*

***Figure 4. Surveillance and monitoring area score, Piedmont Region 2022.***

***Figure 5. Appropriate use of antimicrobials area score, Piedmont Region 2022.***

***Figure 6. Education and training area score, Piedmont Region 2022.***

***Figure 7. Alliance among stakeholders area score, Piedmont Region 2022.***

Considering that the areas “HAI control and prevention” and “Evaluation of the impact and Implementation of the program”include only two standards, scores achieved for these areas were not represented through a spider chart.

***Figure 8. Healthcare-associated infection control and prevention area score, Piedmont Region 2022.***


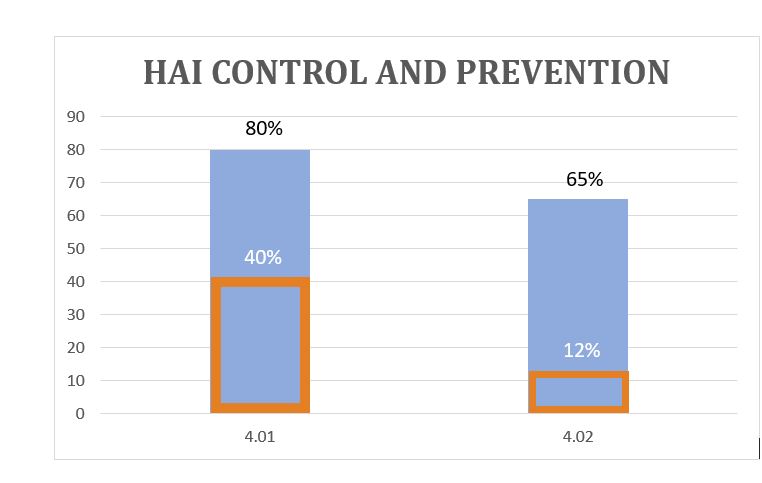


***Figure 9. Evaluation of the impact and implementation of the program area score, Piedmont Region 2022.***


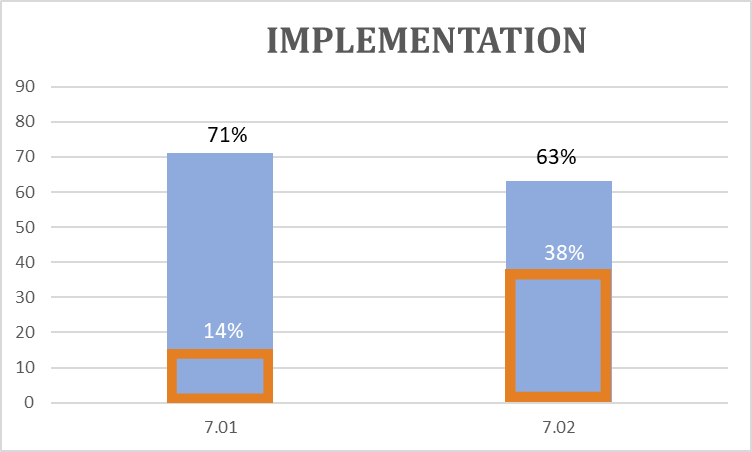


Following are listed the description of all criteria per standard and area (STANDARD 🡪 CRITERIA🡪 DESCRIPTION)

Table A. Minimum Criteria for Governance Area

1.01 1.01.01 The Region/Autonomous Province has formalized the Antimicrobial Resistance Control Plan in a specific document.

1.01 1.01.02 The Antimicrobial Resistance Control Plan explicitly includes interventions for the appropriate use of antimicrobials (antimicrobial stewardship - AMS) in the human sector.

1.01 1.01.03 The Antimicrobial Resistance Control Plan explicitly includes interventions for the surveillance, prevention, and control of healthcare-associated infections (HAIs).

1.01 1.01.04 The Antimicrobial Resistance Control Plan explicitly includes interventions for the appropriate use of antimicrobials and the surveillance, prevention, and control of infections in the veterinary sector (VET).

1.01 1.01.05 The Antimicrobial Resistance Control Plan, with regard to the human sector, provides for interventions aimed at both hospital and community settings.

1.01 1.01.06 The Region/Autonomous Province has identified and formally appointed one or more persons responsible for the Antimicrobial Resistance Control Plan.

1.01 1.01.07 The Antimicrobial Resistance Control Plan defines the specific responsibilities and organizational structures for at least ONE of the Plan’s components (AMS, HAI, VET).

1.02 1.02.01 The Region/Autonomous Province has identified and specified in the Plan the intervention priorities for the appropriate use of antimicrobials in the human sector (AMS).

1.02 1.02.02 The Region/Autonomous Province has identified and specified in the Plan the intervention priorities for the surveillance, prevention, and control of HAIs.

1.02 1.02.03 The Region/Autonomous Province has identified and specified in the Plan the intervention priorities for the appropriate use of antimicrobials and the prevention and control of infections in the veterinary sector (VET).

1.02 1.02.04 The objectives of the Antimicrobial Resistance Control Plan are defined at least every two years based on the identified intervention priorities.

1.02 1.02.05 The Antimicrobial Resistance Control Plan includes specific objectives for the appropriate use of antimicrobials in the human sector (AMS).

1.02 1.02.06 The Antimicrobial Resistance Control Plan includes specific objectives for the prevention and control of HAIs.

1.02 1.02.07 The Antimicrobial Resistance Control Plan includes specific objectives for the appropriate use of antimicrobials and the surveillance, prevention, and control of infections (VET).

1.03 1.03.01 The Region/Autonomous Province involves, in the Antimicrobial Resistance Control Plan—also identifying their respective responsibilities—not only professionals working in the healthcare sector but also those in the livestock sector.

1.03 1.03.02 The Region/Autonomous Province involves, in the Antimicrobial Resistance Control Plan—also identifying their respective responsibilities—not only professionals working in the healthcare sector but also those in the agricultural environmental sector.

1.03 1.03.03 The regional coordination team for the Antimicrobial Resistance Control Plan includes at least one livestock veterinarian (e.g., from the Zooprophylactic Institute).

Table B. Minimum Criteria for Surveillance and Monitoring Area

2.01 2.01.01 The Region/Autonomous Province has formally defined a unique list of ALERT microorganisms to be adopted at the company level.

2.01 2.01.02 The Region/Autonomous Province has established guidelines for the prompt reporting of particularly significant conditions (e.g., microorganisms with unusual resistance profiles, rare infections, or those of particular importance due to severity and contagion).

2.01 2.01.03 The Region/Autonomous Province has established a process for the timely notification of epidemic events in healthcare settings.

2.02 2.02.01 The Region/Autonomous Province has a surveillance system for antimicrobial resistance (at both hospital and community levels) starting from regional public laboratories.

2.02 2.02.02 At least 50% of public Microbiology laboratories are involved in the surveillance system.

2.02 2.02.03 The Region/Autonomous Province participates in national/international antimicrobial resistance surveillance networks (e.g., AR-ISS).

2.03 2.03.01 All regional public laboratories adopt the same reporting methods based on national reporting standards (e.g., AR-ISS, scientific societies, etc.).

2.04 2.04.01 The Region/Autonomous Province produces a report on antimicrobial resistance at least annually at both hospital and community levels.

2.04 2.04.02 The Region/Autonomous Province disseminates the antimicrobial resistance report to all Health Companies/Facilities (e.g., by sending paper copies, email, etc.).

2.04 2.04.03 The report presents data according to national/international indicators (PNCAR, EARS-Net, ECDC, etc.).

2.05 2.05.01 The Region/Autonomous Province periodically analyzes data on antimicrobial consumption (at both hospital and community levels).

2.06 2.06.01 The Region/Autonomous Province produces a report on antimicrobial consumption at least annually at both hospital and community levels.

2.06 2.06.02 Data on antimicrobial consumption are provided at least for each individual Health Company (hospital and community).

2.06 2.06.03 The Region/Autonomous Province disseminates the antimicrobial consumption report to all Health Companies/Facilities (e.g., by sending paper copies, email, etc.).

2.07 2.07.01 PREVALENCE OF HAIs – The system provides for the surveillance of healthcare-associated infections (HAIs) in acute care hospitals through prevalence studies (according to national guidelines or at least every five years).

2.07 2.07.02 PREVALENCE OF HAIs – The system provides for the surveillance of HAIs in residential care facilities through prevalence studies (according to national guidelines or at least every five years).

2.07 2.07.03 PREVALENCE OF HAIs – The Region/Autonomous Province produces a periodic report on HAI surveillance at both hospital and community levels.

2.07 2.07.04 PREVALENCE OF HAIs – At least 50% of acute care hospitals are involved in the surveillance system.

2.07 2.07.05 PREVALENCE OF HAIs – At least 50% of residential care facilities (e.g., nursing homes, etc.) are involved in the surveillance system.

2.08 2.08.01 The system provides for the surveillance of infections in Intensive Care Units.

2.08 2.08.02 The adopted protocol complies with national or international reference documents.

2.09 2.09.01 The system provides for the surveillance of surgical site infections.

2.09 2.09.02 The adopted protocol complies with national or international reference documents.

2.10 2.10.01 In the veterinary field, excluding what is provided by European Directives 2003/99/EC and 652/2013/EC, the Region/Autonomous Province has a continuous monitoring system for antimicrobial resistance in indicator and zoonotic bacteria in animals intended for food production (DPA).

2.10 2.10.02 In the veterinary field, the Region/Autonomous Province has a continuous monitoring system for antimicrobial resistance in pathogenic microorganisms for DPA animals, using the results of antimicrobial susceptibility tests (antibiograms) on clinical isolates produced by microbiological laboratories operating in the Region/Autonomous Province (IZS and private).

2.11 2.11.01 The Region/Autonomous Province has a continuous monitoring system for antimicrobial resistance in companion animals, using the results of antimicrobial susceptibility tests (antibiograms) on clinical isolates produced by microbiological laboratories operating in the Region/Autonomous Province (IZS and private).

2.12 2.12.01 The Region/Autonomous Province has a system for monitoring the use of the National Pharmacovigilance Information System (REV).

2.12 2.12.02 The Region/Autonomous Province promotes the use of ministerial checklists for official pharmacovigilance control.

2.12 2.12.03 The Region/Autonomous Province has adopted the Classy Farm system for categorizing farm risk based on biosecurity indicators, animal welfare, health and production parameters, animal feed, antimicrobial drug consumption, and lesions detected at slaughter for the swine supply chain.

Table C. Minimum Criteria for Appropriate use of antimicrobials Area

3.01 3.01.01 The Region/Autonomous Province promotes the dissemination and implementation of Recommendations for the management of some of the most frequent infectious diseases in the hospital setting.

3.01 3.01.02 The Region/Autonomous Province promotes the dissemination and implementation of Recommendations for the management of some of the most frequent infectious diseases in the community setting.

3.02 3.02.01 The Region/Autonomous Province has adopted policies to contain the consumption of antimicrobials.

3.02 3.02.02 There is a specific regional policy for containing the consumption of at least one of the following classes of antimicrobials: carbapenems, cephalosporins, and quinolones.

3.03 3.03.01 The Region/Autonomous Province has identified and adopted a set of specific evidence-based actions/good practices/bundles for the appropriate use of antimicrobials.

3.03 3.03.02 The Region/Autonomous Province has arranged for public Microbiology Services to be open for at least 12 hours a day and at least 5 days a week.

3.03 3.03.03 The Region/Autonomous Province has adopted a policy that requires all Health Companies/Facilities to promptly communicate (e.g., by phone, visual alert in the electronic medical record, etc.) the positivity of culture tests for MDR microorganisms by the Microbiology Service.

3.04 3.04.01 The Region/Autonomous Province provides guidance to Health Companies/Facilities for the adoption of strategies to assess the appropriateness of antimicrobial prescriptions at the hospital level.

3.05 3.05.01 The Region/Autonomous Province has adopted the available national guidelines for the appropriate use of antimicrobials in food-producing animals.

3.05 3.05.02 The Region/Autonomous Province has adopted specific measures to promote the available national guidelines for the appropriate use of antimicrobials in food-producing animals.

3.06 3.06.01 The Region/Autonomous Province has adopted the available national guidelines for the appropriate use of antimicrobials in companion animals.

3.06 3.06.02 The Region/Autonomous Province has adopted specific measures to promote the available national guidelines for the appropriate use of antimicrobials in companion animals.

Table D. Minimum Criteria for HAI control and prevention Area

4.01 4.01.01 The Region/Autonomous Province has defined and adopted an annual program to implement and support proper hand hygiene.

4.01 4.01.02 The program includes the use of specific indicators (e.g., consumption of alcohol-based hand rub, etc.).

4.02 4.02.01 The Region/Autonomous Province has provided guidance to Health Companies/Facilities regarding the implementation and monitoring of practices for the prevention and control of healthcare-associated infections (HAIs).

4.02 4.02.02 The regional guidelines also call for the involvement of professionals working in the fields of Quality Improvement and Clinical Risk/Patient Safety.

Table E. Minimum Criteria for Education and Training Area

5.01 5.01.01 The person responsible for the Regional Plan for Combating Antimicrobial Resistance has specific skills acquired through specialized training courses (at the international or national level) and/or experience gained during their professional career.

5.01 5.01.02 The staff who are part of the regional coordination team for the Antimicrobial Resistance Control Plan have specific skills acquired through training courses (at the international or national level) and/or experience gained during their professional career.

5.02 5.02.01 The Region/Autonomous Province promotes training on combating antimicrobial resistance and the appropriate use of antimicrobials for healthcare personnel working in public hospital and community facilities and accredited private facilities through regional courses (in-person or online).

5.02 5.02.02 The Region/Autonomous Province provides a training pathway for new hires (doctors, nurses, and healthcare assistants) on combating antimicrobial resistance and the appropriate use of antimicrobials within the first year.

5.02 5.02.03 The Region/Autonomous Province has contributed to the implementation of training activities/courses on combating antimicrobial resistance and the appropriate use of antimicrobials aimed at General Practitioners (MMG) and Pediatricians (PLS) (at least one in the last year).

5.02 5.02.04 The Region/Autonomous Province promotes training on combating antimicrobial resistance and the appropriate use of antimicrobials for pharmacists working in public hospital and community facilities and accredited private facilities through regional courses (in-person or online).

5.02 5.02.05 The Region/Autonomous Province promotes training on combating antimicrobial resistance and the appropriate use of antimicrobials for dentists working in public hospital and community facilities and accredited private facilities through regional courses (in-person or online).

5.03 5.03.01 The Region/Autonomous Province promotes training on the surveillance, prevention, and control of healthcare-associated infections (HAIs) for healthcare personnel working in public hospital and community facilities and accredited private facilities through regional courses (in-person or online).

5.03 5.03.02 The Region/Autonomous Province promotes training on the surveillance, prevention, and control of HAIs for dentists working in public hospital and community facilities and accredited private facilities through regional courses (in-person or online).

5.04 5.04.01 The Region/Autonomous Province promotes training on combating antimicrobial resistance, the appropriate use of antimicrobials, and the surveillance, prevention, and control of infections in the veterinary field, aimed at veterinarians working in the public sector through regional courses (in-person or online).

Table F. Minimum Criteria for Alliance among Stakeholders Area

6.01 6.01.01 The Region/Autonomous Province has planned an information campaign aimed at citizens/patients on the topics of the Antimicrobial Resistance Control Plan.

6.01 6.01.02 The Region/Autonomous Province provides informational materials (including multilingual materials) developed in collaboration with citizens/patients on combating antimicrobial resistance and the appropriate use of antimicrobials, specifically targeted at them.

6.01 6.01.03 The Region/Autonomous Province provides informational materials (including multilingual materials) developed in collaboration with citizens/patients on the prevention and control of healthcare-associated infections (HAIs), specifically targeted at them.

6.02 6.02.01 The Region/Autonomous Province has planned an information campaign aimed at breeders and animal owners on the topics of the Antimicrobial Resistance Control Plan.

6.02 6.02.02 The Region/Autonomous Province provides informational materials developed in collaboration with Breeders’ Associations on combating antimicrobial resistance, the appropriate use of antimicrobials, surveillance, prevention and control of infections in the veterinary field, and the limitation of bacteria transmission between animals and humans.

6.03 6.03.01 In the last two years, the Region/Autonomous Province has carried out at least one initiative for the active involvement of citizens/patients (e.g., meetings with Volunteer Associations, etc.) on combating antimicrobial resistance, the appropriate use of antimicrobials, and the surveillance, prevention, and control of HAIs.

6.04 6.04.01 In the last year, the Region/Autonomous Province has carried out at least one initiative for the involvement of veterinarians working in the private sector on combating antimicrobial resistance, the appropriate use of antimicrobials, and the surveillance, prevention, and control of infections in the veterinary field.

6.04 6.04.02 The Region/Autonomous Province meets at least annually with various representatives from the veterinary, livestock, agricultural, and environmental sectors to define, program, and plan specific initiatives on combating antimicrobial resistance, the appropriate use of antimicrobials, and the surveillance, prevention, and control of infections in the veterinary field.

6.05 6.05.01 In the last year, the Region/Autonomous Province has carried out at least one initiative for the involvement of pharmacists working in community pharmacies on combating antimicrobial resistance and the appropriate use of antimicrobials.

6.06 6.06.01 In the last year, the Region/Autonomous Province has carried out at least one initiative for the involvement of dentists working in the private sector on combating antimicrobial resistance, the appropriate use of antimicrobials, and the surveillance, prevention, and control of HAIs.

6.07 6.07.01 The Region/Autonomous Province meets at least annually with representatives of the Associations/Orders of General Practitioners (MMG) and Pediatricians (PLS) to define and plan specific initiatives on combating antimicrobial resistance, the appropriate use of antimicrobials, and the surveillance, prevention, and control of HAIs.

6.07 6.07.02 In the last year, the Region/Autonomous Province has carried out at least one initiative for the involvement of General Practitioners (MMG), Pediatricians (PLS), Out-of-Hours Doctors, and Prison Medicine Doctors on combating antimicrobial resistance, the appropriate use of antimicrobials, and the surveillance, prevention, and control of HAIs, in collaboration with the Medical Association.

Table G. Minimum Criteria for Evaluation of the impact and Implementation of the program Area

7.01 7.01.01 The Region/Autonomous Province monitors the impact of the Regional Antimicrobial Resistance Control Plan using national indicators (PNCAR indicators).

7.02 7.02.01 The Region/Autonomous Province produces a report (using national PNCAR indicators) on the overall impact of the Regional Antimicrobial Resistance Control Plan at least every two years.

7.02 7.02.02 The Region/Autonomous Province disseminates the report with the impact data of the Regional Antimicrobial Resistance Control Plan to Health Companies/Facilities (e.g., by sending paper materials, email, etc.).

7.02 7.02.03 The report is disseminated to at least 50% of Public Hospital and Community Facilities.

Table H. Additional Criteria for Governance

1.01 1.01.08 The Antimicrobial Resistance Control Plan defines the specific responsibilities and organizational structures for TWO of the Plan’s components (AMS, ICA, VET).

1.01 1.01.09 The Antimicrobial Resistance Control Plan defines the specific responsibilities and organizational structures for ALL THREE components of the Plan (AMS, ICA, VET).

1.01 1.01.10 The Plan provides for the direct involvement of the Regional/Autonomous Province Directorate, through the reporting of results obtained within defined timeframes.

1.01 1.01.11 The Antimicrobial Resistance Control Plan provides for the identification of company representatives for the application of the Plan itself in the various organizational branches (hospital and community).

1.01 1.01.12 The person(s) responsible for the Antimicrobial Resistance Control Plan make use of a technical team for the coordination/monitoring of the Plan that includes all necessary professional roles related to: Prevention Services.

1.01 1.01.13 The person(s) responsible for the Antimicrobial Resistance Control Plan make use of a technical team for the coordination/monitoring of the Plan that includes all necessary professional roles related to: Hospital Care.

1.01 1.01.14 The person(s) responsible for the Antimicrobial Resistance Control Plan make use of a technical team for the coordination/monitoring of the Plan that includes all necessary professional roles related to: Community Care.

1.02 1.02.08 Objectives are defined with the management of Health Companies/Facilities and company representatives for ALL THREE components of the Plan (AMS, ICA, VET).

1.02 1.02.09 The objectives of the Plan are quantitative and are monitored annually for the AMS component of the Plan.

1.02 1.02.10 The objectives of the Plan are quantitative and are monitored annually for the ICA component of the Plan.

1.02 1.02.11 The objectives of the Plan are quantitative and are monitored annually for the VET component of the Plan.

1.02 1.02.12 The indicators used to monitor the objectives are shared with the management of Health Companies/Facilities and with company representatives for the AMS component of the Plan.

1.02 1.02.13 The indicators used to monitor the objectives are shared with the management of Health Companies/Facilities and with company representatives for the ICA component of the Plan.

1.02 1.02.14 The indicators used to monitor the objectives are shared with the management of Health Companies/Facilities and with company representatives for the VET component of the Plan.

1.03 1.03.04 The Antimicrobial Resistance Control Plan provides for specific actions related to the agricultural/environmental sector consistent with the identified objectives.

Table I. Additional Criteria for Surveillance and Monitoring Area

2.01 2.01.04 The Region/Autonomous Province reviews the list of ALERT microorganisms at least biennially.

2.01 2.01.05 The Region/Autonomous Province monitors the adoption of guidelines at the company level at least biennially (e.g., verifying the presence of a company-level procedure).

2.02 2.02.04 All public Microbiology laboratories are involved in the surveillance system.

2.02 2.02.05 The Region/Autonomous Province has a system for monitoring antimicrobial resistance (hospital and territorial levels) that also involves accredited private laboratories.

2.03 2.03.02 The Region/Autonomous Province verifies the presence of a standardized reporting procedure for antimicrobial resistance at the company level at least biennially.

2.03 2.03.03 All accredited private laboratories implement the guidelines for standardized reporting of antimicrobial resistance.

2.04 2.04.04 The report is also distributed to accredited private Structures (e.g., Care Homes, Residential Structures for the Elderly, etc.).

2.04 2.04.05 The report is made available on the regional website.

2.05 2.05.02 The monitoring system integrates with the antimicrobial consumption data in veterinary/zootecnical contexts according to the standards agreed within the national One Health strategy.

2.05 2.05.03 The antimicrobial consumption monitoring system includes data related to accredited private Structures.

2.06 2.06.04 The report includes data on territorial antimicrobial consumption for each functional area of each Associative Form of General Practitioners (MMG) and Pediatricians (PLS) present in the territory.

2.06 2.06.05 The report includes data on antimicrobial consumption of accredited private Structures.

2.06 2.06.06 The report is also distributed to accredited private Structures.

2.06 2.06.07 The report is made available on the regional website.

2.07 2.07.06 PREVALENCE OF HAIs – Over 75% of acute hospital Structures are involved in the surveillance system.

2.07 2.07.07 PREVALENCE OF HAIs – Over 75% of territorial Residential Structures (e.g., RSA, etc.) are involved in the surveillance system.

2.07 2.07.08 PREVALENCE OF HAIs – At least 50% of Accredited Private Structures are involved in the surveillance system.

2.07 2.07.09 PREVALENCE OF HAIs – The Region/Autonomous Province disseminates the surveillance report on HAIs to Hospital Structures (e.g., via paper, email, etc.).

2.07 2.07.10 PREVALENCE OF HAIs – The Region/Autonomous Province disseminates the surveillance report on HAIs to territorial Structures/Services (e.g., via paper, email, etc.).

2.07 2.07.11 PREVALENCE OF HAIs – The report is also distributed to accredited private Structures (e.g., Care Homes, Residential Structures for the Elderly, etc.).

2.07 2.07.12 PREVALENCE OF HAIs – The report is made available on the regional website.

2.10 2.10.04 Using data from the continuous monitoring system of antimicrobial resistance in indicator microorganisms, zoonotic and pathogenic agents in animals, the Region/Autonomous Province promotes the creation of an access system (dashboard) to sensitivity tests to antimicrobials (antibiograms) on clinical isolates produced by microbiological laboratories operating in the Region/Autonomous Province (IZS and private labs).

2.10 2.10.05 The Region/Autonomous Province has a continuous monitoring system for antimicrobial resistance in veterinary medicine in animals DPA, which uses a dedicated study, identifying the sample size, farms (animal species and farm type), animals (zootecnical category), from which to collect samples for indicator bacteria for susceptibility testing (antibiograms).

2.10 2.10.06 The Region/Autonomous Province disseminates the results of the continuous monitoring of antimicrobial resistance in indicator microorganisms, zoonotic and pathogenic agents in animals DPA to healthcare operators in the relevant Structures and sector operators through at least one mode among those indicated (sending physical material to individual Structures, regional website, company website, interactive dashboard).

2.11 2.11.02 The Region/Autonomous Province produces, at least annually, a report on antimicrobial resistance in companion animals.

2.11 2.11.03 Using data from the continuous monitoring system of antimicrobial resistance in companion animals, the Region/Autonomous Province creates a continuous access system (dashboard) for sensitivity tests to antimicrobials (antibiograms) on clinical isolates produced by microbiological laboratories operating in the Region/Autonomous Province (IZS and private labs).

2.11 2.11.04 The reports are disseminated to healthcare operators in the relevant Structures and sector operators through at least one mode among those indicated (sending physical material to individual Structures, regional website, company website, interactive dashboard).

2.12 2.12.04 The Region/Autonomous Province has adopted the Classy farm system for categorizing the risk of farms based on biosafety indicators, animal welfare, health and productivity parameters, animal feed, and consumption for the poultry supply chain.

2.12 2.12.05 The Region/Autonomous Province has adopted the Classy farm system for categorizing the risk of farms based on biosafety indicators, animal welfare, health and productivity parameters, animal feed, and consumption for the beef cattle supply chain.

2.12 2.12.06 The Region/Autonomous Province uses antimicrobial prescription indicators for individual production chains to produce a descriptive report on antimicrobial prescription.

2.12 2.12.07 The report is available to all operators (both company and private).

Table J. Additional Criteria for Appropriate use of antimicrobials Area

3.01 3.01.03 The Region/Autonomous Province has produced support materials for prescribers (e.g., antimicrobial use handbooks, booklets, etc.).

3.01 3.01.04 Regional recommendations are available for the management of Urinary Tract Infections.

3.01 3.01.05 Regional recommendations are available for the management of Bacterial Pneumonias.

3.01 3.01.06 Regional recommendations are available for the management of Skin and Soft Tissue Infections.

3.01 3.01.07 Regional recommendations are available for the empirical treatment of Abdominal Infections.

3.01 3.01.08 Regional recommendations are available for the empirical treatment of Bacterial Sepsis.

3.01 3.01.09 Regional recommendations are available for initiating empirical antimicrobial treatment based on the correct interpretation of laboratory markers of inflammation/infection.

3.01 3.01.10 Regional recommendations are available for initiating empirical treatment of infections in specific types of patients (immunocompromised, hemodialysis patients, etc.).

3.01 3.01.11 Regional recommendations are available for Antimicrobial Prophylaxis in Surgery.

3.01 3.01.12 Regional recommendations are available to all healthcare personnel, both hospital and community-based, through at least one of the following methods: sending printed materials, regional website, company website.

3.01 3.01.13 The Region/Autonomous Province reviews/updates the recommendations at least every two years.

3.01 3.01.14 The Region/Autonomous Province instructs Health Companies/Facilities to verify at least biennially the actual application of at least one recommendation by healthcare professionals and requires periodic submission of data.

3.02 3.02.03 The Region/Autonomous Province has adopted policies to contain the consumption of certain classes of antimicrobials specifically aimed at pediatric patients.

3.02 3.02.04 There is a specific regional policy for containing the consumption of more than one of the following classes of antimicrobials: carbapenems, cephalosporins, and quinolones.

3.02 3.02.05 The Region/Autonomous Province instructs Health Companies/Facilities to verify compliance with the policy on antimicrobial prescription containment annually.

3.03 3.03.04 The set of actions/good practices/bundles is reviewed at least every two years.

3.03 3.03.05 The Region/Autonomous Province instructs Health Companies/Facilities to verify at least biennially the adherence of healthcare personnel to actions/good practices/bundles.

3.03 3.03.06 The Region/Autonomous Province disseminates to Health Companies/Facilities the results of monitoring compliance with actions/good practices/bundles (e.g., via reports).

3.03 3.03.07 The Region/Autonomous Province has arranged for the prompt availability of public Microbiology Services during night hours and holidays.

3.03 3.03.08 The Region/Autonomous Province has arranged for public Microbiology Services to be open 24 hours a day, 7 days a week.

3.03 3.03.09 The Region/Autonomous Province has adopted a policy that requires all Health Companies/Facilities to communicate by phone the positivity of blood culture and cerebrospinal fluid culture results from the Microbiology Service.

3.04 3.04.02 The Region/Autonomous Province provides guidance to Health Companies/Facilities for adopting strategies to assess the appropriateness of antimicrobial prescriptions at the community level.

3.04 3.04.03 Accredited Private Facilities (e.g., Care Homes, Residential Structures for the Elderly, etc.) are also involved in the evaluation process.

3.05 3.05.03 The Region/Autonomous Province has adopted national guidelines for the appropriate use of antimicrobials in pig farming.

3.05 3.05.04 The Region/Autonomous Province has adopted national guidelines for the appropriate use of antimicrobials in dairy cattle farming.

3.05 3.05.05 The Region/Autonomous Province has adopted national guidelines for the appropriate use of antimicrobials for other species, when available.

3.05 3.05.06 The Region/Autonomous Province has implemented a monitoring system based on data available through the Electronic Veterinary Prescription to verify antimicrobial use.

3.05 3.05.07 The Region/Autonomous Province has adopted specific measures to promote the involvement of Universities, Professional Organizations, Experimental Zooprophylactic Institutes, and Trade Associations in the program.

3.06 3.06.03 The Region/Autonomous Province has adopted tools to monitor the use of national guidelines for the appropriate use of antimicrobials in companion animals (matching prescriptions to guideline recommendations).

3.06 3.06.04 The Region/Autonomous Province promotes the application of national guidelines for the appropriate use of antimicrobials in companion animals by operators at least every three years.

3.06 3.06.05 The Region/Autonomous Province uses electronic veterinary prescription data to verify the compliance of operators (prescribers) with national guidelines for the appropriate use of antimicrobials in companion animals.

Table K. Additional Criteria for HAI control and prevention Area

4.01 4.01.03 The Region/Autonomous Province produces an annual report with the results of indicators measuring the adherence of Health Companies/Facilities to programs implemented in support of proper hand hygiene.

4.01 4.01.04 The Region/Autonomous Province disseminates the report to Health Companies/Facilities on the results of hand hygiene indicators (e.g., through regional events, email, etc.).

4.01 4.01.05 Accredited Private Facilities are also involved in the program.

4.02 4.02.02 The regional guidelines also call for the involvement of professionals working in the fields of Quality Improvement and Clinical Risk/Patient Safety.

4.02 4.02.03 The Region/Autonomous Province has provided guidance to Health Companies/Facilities to encourage healthcare personnel to adhere to standard precautions.

4.02 4.02.04 The Region/Autonomous Province has provided guidance to Health Companies/Facilities to encourage healthcare personnel to adhere to precautions for the prevention of infections transmitted by contact, airborne, and droplet routes.

4.02 4.02.05 The Region/Autonomous Province has provided guidance to Health Companies/Facilities to encourage the adoption of actions, bundles, or best practices for the prevention of device/invasive procedure-related infections (surgical site, CVC-related bloodstream infections, VAP, HAP, etc.).

4.02 4.02.06 The Region/Autonomous Province has provided guidance to Health Companies/Facilities for the management of epidemic infectious events.

4.02 4.02.07 The Region/Autonomous Province has provided guidance to Health Companies/Facilities for the management of sentinel (ALERT) microorganisms.

4.02 4.02.08 The Region/Autonomous Province has provided guidance to Health Companies/Facilities for the management of patients colonized/infected with MDR microorganisms (CPE, CR-Pseudomonas, CR-Acinetobacter, MRSA, VRE, etc.).

4.02 4.02.09 The Region/Autonomous Province has provided guidance to Health Companies/Facilities for the protection from infectious risk and safety of healthcare workers.

4.02 4.02.10 The Region/Autonomous Province has provided guidance to Health Companies/Facilities for hygienic-sanitary procedures (disinfection and sterilization, infectious waste, etc.).

4.02 4.02.11 The Region/Autonomous Province instructs Health Companies/Facilities to verify at least biennially the actual adherence to at least one guideline on practices for the prevention and control of healthcare-associated infections (HAIs) and requires periodic submission of data.

4.02 4.02.12 The Region/Autonomous Province disseminates to Health Companies/Facilities the results of monitoring adherence to guidelines on practices for the prevention and control of HAIs.

4.02 4.02.13 Accredited Private Facilities are also involved in the program.

4.02 4.02.14 The Region/Autonomous Province provides specific guidance to Health Companies/Facilities for the adoption of multimodal strategies for the implementation of the Surveillance, Prevention, and Control Program for HAIs.

4.02 4.02.15 The guidance provided includes interventions/activities related to two or more of the following areas: Organizational and/or system changes, Education and Training, Monitoring and feedback of data, Communication and educational reinforcement, Cultural change and development of a safety-oriented culture

4.02 4.02.16 The guidance provided includes the use of bundles and/or checklists.

4.02 4.02.17 The Region/Autonomous Province instructs Health Companies/Facilities to verify at least annually the effectiveness of the adopted multimodal strategies.

4.02 4.02.03 The Region/Autonomous Province has provided guidance to Health Companies/Facilities to encourage healthcare personnel to adhere to standard precautions.

Table L. Additional Criteria for for Education and Training Area

5.01 5.01.03 The healthcare personnel of the Health Companies/Facilities responsible for the Antimicrobial Resistance Control Plan (link-professional) have specific skills acquired through participation in training courses and/or on-the-job training activities organized at the company and/or regional level (at least one carried out in the last three years).

5.02 5.02.07 In the last three years, the Region/Autonomous Province has recorded an increase in the number of operators (doctors, nurses, and healthcare assistants) participating in the regional course.

5.02 5.02.08 In the last three years, the Region/Autonomous Province has recorded an increase in the number of General Practitioners (MMG) and Pediatricians (PLS) participating in training activities/courses on the topic.

5.02 5.02.09 The Region/Autonomous Province has contributed to the implementation of training activities/courses on the topic of combating antimicrobial resistance and the appropriate use of antimicrobials in collaboration with the Order of Pharmacists aimed at Pharmacists working in community pharmacies (at least one in the last year).

5.02 5.02.10 In the last three years, the Region/Autonomous Province has recorded an increase in the number of Pharmacists working in Hospital and community Facilities participating in the regional course.

5.02 5.02.11 In the last three years, the Region/Autonomous Province has recorded an increase in the number of Pharmacists working in community pharmacies participating in training activities/courses on the topic.

5.02 5.03.03 The Region/Autonomous Province has contributed to the implementation of training activities/courses on the topic of surveillance, prevention, and control of healthcare-associated infections, aimed at General Practitioners (MMG) and Pediatricians (PLS) (at least one in the last year).

5.03 5.03.04 In the last three years, the Region/Autonomous Province has recorded an increase in the number of operators (doctors, nurses, and healthcare assistants) participating in the regional course.

5.03 5.03.05 The Region/Autonomous Province provides a training path for new hires (doctors, nurses, and healthcare assistants) on the topic of surveillance, prevention, and control of healthcare-associated infections within the first year.

5.03 5.03.06 In the last three years, the Region/Autonomous Province has recorded an increase in the number of General Practitioners (MMG) and Pediatricians (PLS) participating in training activities/courses on the topic of surveillance, prevention, and control of healthcare-associated infections.

5.04 5.04.02 The Region/Autonomous Province has contributed to the implementation of training activities/courses on the topics of combating antimicrobial resistance, appropriate use of antimicrobials, and surveillance, prevention, and control of infections in the veterinary field, aimed at Veterinarians working in the private sector (at least one in the last year), in collaboration with the Order of Veterinarians.

5.04 5.04.03 In the last three years, the Region/Autonomous Province has seen an increase in the number of Veterinarians working in the public sector participating in the regional course.

5.04 5.04.04 In the last three years, the Region/Autonomous Province has seen an increase in the number of Veterinarians working in the private sector participating in training activities/courses on the topic.

Table M. Additional Criteria for Alliance among Stakeholders Area

6.01 6.01.04 The informational material has been distributed to all branches of the Regional Health Service (Hospital Structures, Territorial Structures, Pharmacies, General Practitioner Clinics, etc.)

6.01 6.01.05 The informational material is easily accessible to citizens/patients also on the regional website

6.01 6.01.06 Healthcare personnel have been informed about the content of the informational material and the methods for distributing it to citizens/patients

6.01 6.01.07 The information campaign aimed at citizens includes the use of specific information dissemination tools (TV spots, radio spots, billboards, social media, etc.)

6.01 6.01.08 The Region/Autonomous Province makes available to citizens/patients the data contained in the reports relating to antimicrobial consumption and the prevalence and incidence of healthcare-associated infections

6.01 6.01.09 The Region/Autonomous Province has formalized agreements with the Universities present in the Region/Autonomous Province for the inclusion in the curricula of health degree courses of training programs on the topic of combating antimicrobial resistance and the appropriate use of antimicrobials and surveillance, prevention, and control of healthcare-associated infections

6.01 6.01.10 The Region/Autonomous Province has an education/information program specifically aimed at particular types of patients and their families (e.g., immunocompromised subjects, colonized by MDR microorganisms, transplant recipients, etc.) aimed at reducing the risk of healthcare-associated infections

6.01 6.01.04 The informational material has been distributed to all branches of the Regional Health Service (Hospital Structures, Territorial Structures, Pharmacies, General Practitioner Clinics, etc.)

6.02 6.02.03 The Region/Autonomous Province has planned an information campaign aimed at farmers on the topics of the Antimicrobial Resistance Control Plan

6.02 6.02.04 The Region/Autonomous Province has prepared informational material (also multilingual) for the public, breeders, and farmers freely available on the regional website

6.02 6.02.05 Veterinary healthcare personnel have been informed about the content of the informational material and the methods for distributing it to breeders, animal owners, and farmers

6.03 6.03.02 In the last two years, the Region/Autonomous Province has carried out more than one initiative for the involvement of citizens/patients on the topic

6.03 6.03.03 The Region/Autonomous Province meets at least biennially with Citizens’/Patients’ Associations for the definition, programming, and planning of specific initiatives on the topics of combating antimicrobial resistance, appropriate use of antimicrobials, and surveillance, prevention, and control of healthcare-associated infections

6.03 6.03.04 The Region/Autonomous Province meets at least biennially with Third Sector Associations for the definition and planning of initiatives for the active involvement of citizens/patients

6.04 6.04.03 In the last year, the Region/Autonomous Province has carried out more than one initiative for the involvement of Veterinarians working in the private sector on the topics of combating antimicrobial resistance, appropriate use of antimicrobials, and surveillance, prevention, and control of infections in the veterinary field

6.04 6.04.04 In the last year, the Region/Autonomous Province has carried out more than one initiative for the involvement of breeders on the topics of combating antimicrobial resistance, appropriate use of antimicrobials, and surveillance, prevention, and control of infections in the veterinary field

6.05 6.05.02 In the last year, the Region/Autonomous Province has carried out more than one initiative for the involvement of Pharmacists working in community pharmacies on the topics of combating antimicrobial resistance and appropriate use of antimicrobials

6.05 6.05.03 The Region/Autonomous Province meets at least annually with representatives of Pharmacists’ Associations/Orders for the definition and planning of specific initiatives on the topics of combating antimicrobial resistance and appropriate use of antimicrobials

6.06 6.06.02 In the last year, the Region/Autonomous Province has carried out more than one initiative for the involvement of Dentists working in the private sector on the topics of combating antimicrobial resistance, appropriate use of antimicrobials, and surveillance, prevention, and control of healthcare-associated infections

6.06 6.06.03 The Region/Autonomous Province meets at least annually with representatives of Dentists’ Associations/Orders for the definition and planning of specific initiatives on the topics of combating antimicrobial resistance, appropriate use of antimicrobials, and surveillance, prevention, and control of healthcare-associated infections

6.07 6.07.03 In the last year, the Region/Autonomous Province has carried out more than one initiative for the involvement of General Practitioners and Pediatricians on the topics of combating antimicrobial resistance, appropriate use of antimicrobials, and surveillance, prevention, and control of healthcare-associated infections

6.07 6.07.04 The Region/Autonomous Province meets at least annually with representatives of General Practitioners’ and Pediatricians’ Associations/Orders for the sharing of data relating to antimicrobial consumption, bacterial resistance, and prescribing appropriateness, prevention, and control of healthcare-associated infections

Table N. Additional Criteria for Evaluation of the impact and Implementation of the program Area

7.01 7.01.02 Impact data are shared with the Management of Health Companies/Facilities at least annually.

7.01 7.01.03 The Region/Autonomous Province, based on the impact data, carries out an analysis of possible critical issues/barriers and strengths (e.g., aspects related to personnel or technological and instrumental equipment, organizational aspects, etc.) that have hindered or favored the achievement of the set objectives.

7.01 7.01.04 The Region/Autonomous Province defines a specific program of activities for the removal/management of the identified critical issues/barriers.

7.01 7.01.05 The Region/Autonomous Province defines specific actions for the enhancement of the identified strengths.

7.02 7.02.04 The report is disseminated to over 75% of Public Hospital and Territorial Structures.

7.02 7.02.05 The report is also disseminated to Accredited Private Structures.

7.02 7.02.06 The report is made available on the regional website.

7.02 7.02.07 The report provided to individual Health Companies/Facilities indicates the data at the level of each individual Hospital Structure and Territorial Structure/Service.

7.02 7.02.08 The Region/Autonomous Province has provided one or more methods for sharing and/or critical analysis of the results with the Management of Companies/Facilities present in the Region/Autonomous Province.
